# Supplementary material for: De Ritis Ratio to Predict Clinical Outcomes of Intermediate- and High-Risk Pulmonary Embolisms
Source: J Clin Med. 2024 Apr 4;13(7):2104. doi: 10.3390/jcm13072104 (PMC11012845; doi:10.3390/jcm13072104)
Supplement: Supplementary file 1 [file jcm-13-02104-s001.zip › jcm-2715696-supplementary.pdf]

Table S1 Missing data

Variable	Number missing	Percentage missing
**BNP**	48	20.8
**Troponin**	25	9.2

Abbreviations: BNP—B-type natriuretic peptide; CVP—central venous pressure; RVSP—right ventricular systolic pressure.

Figure S1 Directed acyclic graph of covariates

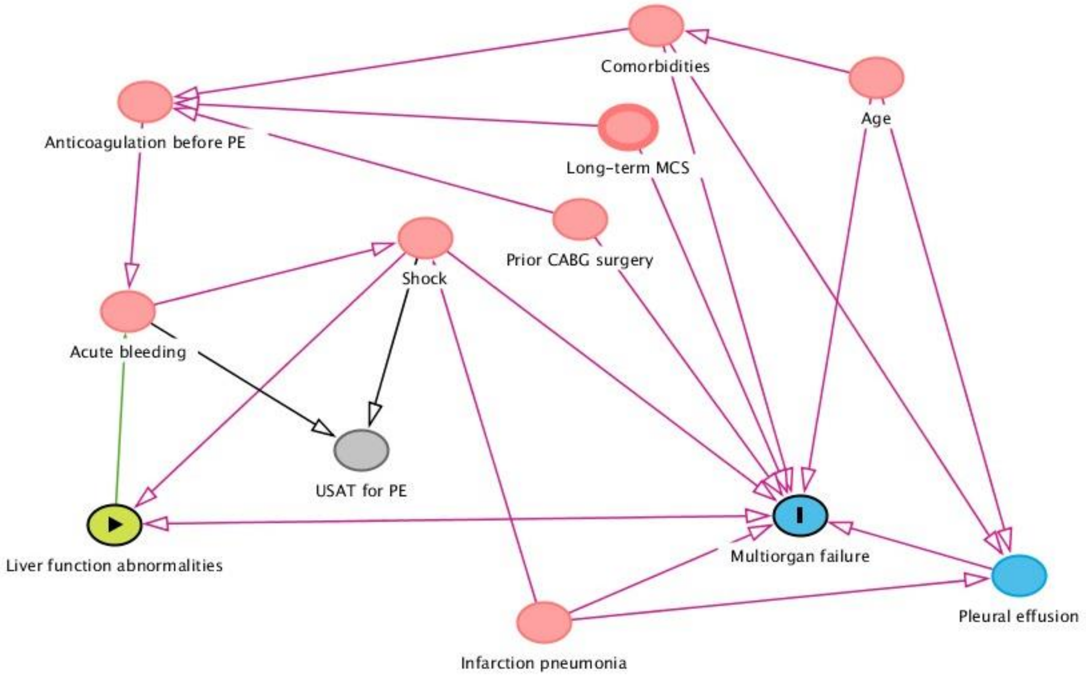

Directed acyclic graph (DAG) based on the influence of liver function abnormalities on multiorgan failure. Gray consists of other outcomes, green consists of (ancestor of) exposure, blue of (ancestor of) outcome and pink of ancestor of exposure and outcome, and thus as confounder.

Comorbidities include arterial hypertension, coronary artery disease, chronic left heart failure, precerebral stenosis, chronic obstructive pulmonary disease, diabetes mellitus type 2, obesity. and chronic kidney disease.

Abbreviations: CABG—coronary artery bypass graft; MCS—Mechanical circulatory support; PE—pulmonary embolism; USAT—ultrasound assisted thrombolysis.

Table S2 Covariate balance after inverse probability weighting

Covariate	Normal vs. Abnormal AST/ALT-ratio	
	Weighted SMD	Unweighted SMD
**Intermediate low-risk**	0.071	0.411
**Intermediate high-risk**	0.088	0.196
**High-risk**	0.024	0.755
**Age**	0.260	0.314
**Female gender**	0.167	0.003
**Long-term MCS**	0.188	0.198
**USAT for PE**	0.068	0.622
**Arterial hypertension**	0.081	0.106
**Coronary artery disease**	0.019	0.316
**Chronic left heart failure**	0.298	0.561
**COPD**	0.141	0.151
**Pleural effusion**	0.028	0.077
**Anticoagulation before PE**	0.017	0.037
**Prior CABG-surgery**	0.048	0.132
**Diabetes mellitus type 2**	0.053	0.211

**Obesity**	0.019	0.127
**Chronic kidney disease**	0.019	0.001
**Infarction pneumonia**	0.032	0.025
**Shock**	0.024	0.755

Abbreviations: ALT—alanine transaminase; AST—aspartate transaminase; CABG—coronary artery bypass graft; COPD—chronic obstructive pulmonary disease; CVP—central venous pressure; LOS—length of stay; MCS—Mechanical circulatory support; PE—pulmonary embolism; RVSP—right ventricular systolic pressure; SMD—standardized mean difference; USAT—ultrasound assisted thrombolysis.

Figure S2 Covariate balance after inverse probability weighting

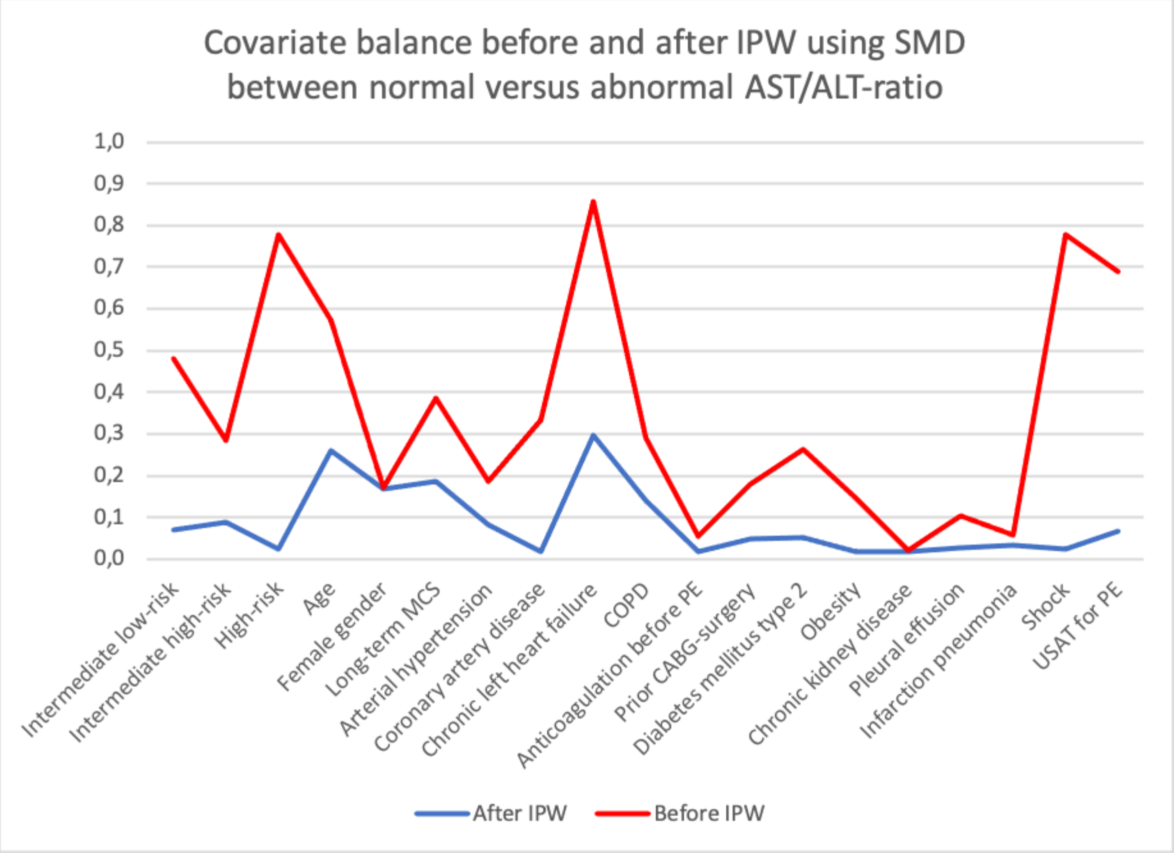

Abbreviations: ALT—alanine transaminase; AST—aspartate transaminase; CABG—coronary artery bypass graft; COPD—chronic obstructive pulmonary disease; CVP—central venous pressure; LOS—length of stay; MCS—Mechanical circulatory support; PE—pulmonary embolism; RVSP—right ventricular systolic pressure; SMD—standardized mean difference; USAT—ultrasound-assisted thrombolysis.
